# Association of housing adaptation services with the prevention of care needs level deterioration for older adults with frailty in Japan: a retrospective cohort study

**DOI:** 10.1186/s12913-023-09890-x

**Published:** 2023-08-29

**Authors:** Rumiko Tsuchiya-Ito, Shota Hamada, Masao Iwagami, Ayako Ninomiya, Tomoaki Ishibashi

**Affiliations:** 1 Research Department, Institute for Health Economics and Policy, Association for Health Economics Research and Social Insurance and Welfare, Tokyu Toranomon Bldg, 1-21-19 Toranomon, Minato-ku, Tokyo, 105-0001 Japan; 2 Dia Foundation for Research on Ageing Societies, Tokyo, Japan; 3 Department of Health Services Research, Institute of Medicine, University of Tsukuba, Tsukuba, Japan; 4 Department of Home Care Medicine, Graduate School of Medicine, The University of Tokyo, Tokyo, Japan; 5 Faculty of Nursing, Josai International University, Chiba, Japan

**Keywords:** Older adults, Claims data, Building environment, Disability, Long-term care

## Abstract

**Background:**

Housing adaptations are aimed at minimizing the mismatch between older adults’ functional limitations and their building environments. We examined the association of housing adaptations with the prevention of care needs level deterioration among older adults with frailty in Japan.

**Methods:**

The subjects comprised individuals who were first certified as having care support levels (defined as frail, the lowest two of seven care needs levels) under the public long-term care insurance systems between April 2015 and September 2016 from a municipality close to Tokyo. The implementation of housing adaptations was evaluated in the first six months of care support certification. Survival analysis with Cox proportional hazards model was performed to examine the association between housing adaptations and at least one care needs level deterioration, adjusting for age, sex, household income level, certified care support levels, cognitive function, instrumental activities of daily living, and the utilization of preventive care services (designed not to progress disabilities). We further examined the differences in the association of the housing adaptation amount by categorizing the subjects into the maximum cost group (USD 1,345–1,513) or not the maximum cost group (< USD 1,345). All the subjects were followed until the earliest of deterioration in care needs level, deaths, moving out of the municipality, or March 2018.

**Results:**

Among 796 older adults, 283 (35.6%) implemented housing adaptations. The incidence of care needs level deterioration was 19.3/1000 person-month of older adults who implemented housing adaptations, whereas 31.9/1000 person-month of those who did not. The adjusted hazard ratio (aHR) of care needs level deterioration was 0.69 (95% confidence interval (CI): 0.51–0.93). The aHRs were 0.51 (95% CI: 0.31–0.82) and 0.78 (95% CI: 0.57–1.07) in the maximum and not maximum cost groups, respectively.

**Conclusions:**

Housing adaptations may prevent care needs level deterioration of older adults with frailty. Policymakers and health professionals should deliver housing adaptations for older adults at risk of increasing care needs.

## Background

The housing environments of older adults, especially those with disabilities, must be able to compensate for losses of capacity, supporting their independence and well-being [1]. Housing adaptations are structural changes made to a building such as widening door and device installations, such as handrails [2]. Japanese long-term care insurance system delivers six types of housing adaptations: installation of handrails, elimination of height differences, change of floor materials, change of doors, change of lavatory basins, and other adaptations accompanying these five types of adaptations [3]. They remove environmental barriers to support or compensate for the loss of functional capacity and minimize the mismatch between functional limitations and environments [2, 4] and improve or maintain older adults’ abilities to perform activities of daily living (ADL) [5]. They also affect health-related outcomes, including preventing fall-related injuries, [6] and maintaining the quality of life [7].

One other important factor to measure the effects of housing adaptions should be the time required for care. An increase in the time required for care may impose greater burdens on long-term care systems and informal care. However, the effects of housing adaptations on the time required for care were inconclusive [8, 9]. Additionally, previous studies have some limitations due to the before/after comparison design [8] and possible confounding by subjects’ characteristics [9]. Moreover, it is important to consider the total amount of housing adaptations not just types of housing adaptation [2, 10, 11] because the accessibility and safety within building environments are ensured only when multiple adaptations work as a whole.

Additionally, focusing on the older adults with similar states of disabilities would be important because the purposes of housing adaptations vary depending on beneficiaries’ disabilities [12]. Under the Japanese long-term care insurance system, long-term care needs levels are determined based on the standard time required for care and certified across seven categories: care support levels 1 to 2 (indicative of the need for preventive care) and care needs levels 1 to 5 (indicative of the need for long-term care), with higher levels signifying the greater need for care. Older adults certified as care support levels, as a state of increased vulnerability to adverse outcomes, [13] could be regarded as those with frailty [14]. Care support levels do not need long-term care services but are at risk of functional deterioration [14]. Care needs level deterioration means that there is an extension of the required care time and is a feasible indicator for detecting changes in the care needs. Therefore, this study examined the association of housing adaptations with the care needs level deterioration among older adults with frailty.

## Methods

### Data sources and design

We conducted a retrospective cohort study using administrative data obtained from a municipality close to Tokyo. The population of this city was about 490,000, and older adults aged 65 years or older was 24.2% in 2015, which is slightly lower than the national average of 26.7% [15]. The data included long-term care insurance eligibility, care needs certification for long-term care insurance service use, insurance premium levels, and long-term care claims data. We extracted information on the date of care needs certification and care needs levels from the long-term care insurance eligibility as well as on each individual’s degrees of physical and cognitive impairment from the care needs certification data [16]. We used long-term care insurance claims data to identify the expenditures, amounts, and types of individual preventive care services that each recipient used. Long-term insurance claims data is stored monthly by the municipality. Insurance premium levels are determined based on individual and household-level taxation, and we used those as a proxy of household income. We used these datasets following an agreement from a collaborative research project between the municipality and the Dia foundation for research on ageing societies.

### Study subjects

This study included individuals certified as having care support level 1 or 2 for the first time between April 2015 and September 2016 and utilized any preventive care services within six months after certifications. We used landmark analysis [17] to prevent immortal time bias; [18] thus, we used the first six months after the certifications to determine the status of housing adaptations, and we started to follow the implemented and not implemented housing adaptation groups six months after the certifications. We checked whether the current certification was given for the first time using care needs certification data after April 2007. The duration of six months to determine the implementation of housing adaptations was defined because the period for renewal in newly certified persons is six months basically and a maximum of 12 months [14]. Moreover, in another study, 80% of care support levels’ older adults who conducted housing adaptations received housing adaptation in the first six months after their certifications [19].

### Independent variable

Housing adaptations during the first six months after the care needs certifications were used as the independent variable. Long-term care beneficiaries cannot use homecare services over the upper limits of service costs depending on each certified levels but the housing adaptations can be used multiple times as long as the accumulated cost is within JPY 200,000 (USD 1,681) in individual’s life [3]. The currency was converted from Japanese yen to US dollars using the exchange rate in April 2015 (USD 1 = JPY 118.9) [20]. The copayment rate is 10 or 20% depending on the household or individual income, and the cost of housing adaptation in this study was not including out-of-pocket payments but only paid by long-term care insurance, which is 80 or 90% of housing adaptation cost. We could not recognize each subject’s copayment from the claims database used for this study. To determine the independent variable, we summed the costs if the subject used housing adaptations multiple times during the first six months. Additionally, to examine how the different amounts of housing adaptations were associated with the care needs level deterioration, we divided the housing adaptations group according to the maximum cost group (USD 1,345–1,513) or not maximum cost group (< USD 1,345). This threshold was determined whether the subject used the maximum housing adaptation costs or not: the cost was USD 1,345 for individuals with 20% copayments and USD 1,513 for individuals with 10% copayments.

### Dependent variable

The dependent variable was at least one level of care needs level deterioration among the seven levels of care needs certification. Long-term care needs certifications are evaluated when the valid period of certification is expired, when beneficiaries hope to change their certification level, or medical or long-term care professional detect their unmet needs for long-term care.

### Statistical analysis

We conducted a χ^2^ test to compare individual characteristics by the implementation of housing adaptations, and we illustrated Kaplan–Meier plots of care needs deterioration for housing adaptations. Subsequently, we estimated the likelihood of care needs level deterioration related to housing adaptation implementation with the Cox proportional hazard model adjusted for covariates. Furthermore, the likelihood of care needs level deterioration was estimated by the total cost of housing adaptations divided into maximum or not. The covariates were age (65–74/75–84/≥ 85 years), sex (female/male), household income level (low/middle-to-high), certified care levels (care support level 1/ 2), cognitive function, instrumental activities of daily living (IADL), and the utilization of preventive care services based on previous works of literature (Table 1) [19, 21–24]. We selected five items as IADL by comparing the assessment of long-term care certifications with Lawton’s IADL scale [25]. That is, *Shopping* from the assessment of long-term care certifications was selected as “Shopping” in Lawton’s IADL scale. Similarly, *Cooking* was selected as “Food Preparation,” *Going outside* was selected as “Mode of Transportation,” *Taking medicine* was selected as “Responsibility for own medication,” and *Money management* was selected as “Ability to Handle Finance.” “Ability to use telephone,” “Housekeeping,” and “Laundry” in Lawton’s IADL scale were not included because there were no corresponding items in the assessment of long-term care certifications. We considered using basic activities of daily living (BADL) and IADL as covariates but excluded BADL because few of those subjects had disabilities in BADL. The results of the survival analysis revealed adjusted hazard ratios (aHRs) and 95% confidence intervals (95% CIs). Older adults who moved out of the municipality or died during the follow-up period were censored. The proportional hazards assumption was inspected using double logarithms plot. In addition, we conducted a sensitivity analysis to evaluate the implementation of housing adaptations in the first 12 months to examine the robustness of the evaluation in the first six months of care support certification. Statistical analyses were conducted using IBM SPSS Statistics ver.25, and the significance threshold was set at *p* = 0.05 (two-tailed).

**Table 1:** Covariates

Indices	Categories
Age	65–74/75–84/≥ 85 years
Sex	female/male
Household income	‧ low (Long-term care insurance premium levels 1 to 4; exemption from residential tax) ‧ middle-to-high (Long-term care insurance premium levels 5 to 18; not exempted from residential tax)
Cognitive function [16]	‧ independent (independent) ‧ mild (Rank I: A person who has some type of dementia, but is mostly independent in terms of daily activities at home and in society) ‧ moderate (Rank II: A person who has some symptoms, behaviors, and communication difficulties that disrupt daily life, but can live independently if someone is watching over them or higher ranks)
IADL	*Going outside*: at least once a week/ less than once a week but at least once a month/less than once a month
	*Taking medicine*: independent/needs help
	S*hopping*: independent/needs help
	*Money management*: independent/partially needs help/totally needs help
	C*ooking*: independent/partially needs help/totally needs help
Preventive care services ^a)^	used/not used

^a)^ Preventive care services include home-visit services (home help, bathing, nursing, and rehabilitation), day services (supporting daily housework and activities) or day care services (rehabilitation), short-stay services, physician/dentist home visits, renting or purchasing assistive devices, community-based multiple care services, and long-term care needs prevention and comprehensive livelihood support services [33, 34]. Assistive devices (renting/purchasing) were not included because of the multicollinearity between housing adaptations and the utilization of assistive devices

## Results

Among the long-term care beneficiaries with certified care support levels (n = 2,137), 966 subjects utilized preventive care services within six months after certifications. We selected people who utilized preventive care services because around half of older adults with certified care support levels did not urgently need them but had applied in case the person suddenly needed preventive care services [26, 27]. We excluded those aged < 65 years (n = 27), and public assistance recipients (n = 47). Additionally, those who died or moved out of the municipality (n = 26), those who were admitted to long-term care facilities or group homes (n = 8), and those whose care needs levels deteriorated (n = 62) during the six-month baseline period were excluded. Consequently, 796 older adults were selected for further analyses.

Individual characteristics of older adults with certified care support levels are shown according to housing adaptation implementation in Table 2. In total, 283 (35.6%) older adults implemented housing adaptations during the first six months after the certifications. Those who received housing adaptations were less likely to have certified care support level 1 (compared with support level 2) and to use preventive care services.

**Table 2:** Characteristics of study subjects according to implementation of housing adaptations for older adults certified for care support levels

	Housing adaptations		*p*-value ^a)^
	Not implemented	Implemented	
**N (% of all)**	513 (64.4)	283 (35.6)	
**Age (years)**			
65 − 74	112 (21.8)	65 (23.0)	0.529
75 − 84	276 (53.8)	159 (56.2)	
≥85	125 (24.4)	59 (20.8)	
**Sex**			
Female	326 (63.5)	180 (63.6)	0.987
Male	187 (36.5)	103 (36.4)	
**Household income**			
Low	287 (55.9)	149 (52.7)	0.371
Middle-to-High	226 (44.1)	134 (47.3)	
**Certified levels**			
Care support level 1	292 (56.9)	136 (48.1)	0.016
Care support level 2	221 (43.1)	147 (51.9)	
**Cognitive function**			
Independent	231 (45.0)	136 (48.1)	0.710
Mildly declined (rank I)	229 (44.6)	120 (42.4)	
Moderately declined (rank II or higher)	53 (10.3)	27 (9.5)	
**IADL**			
*Going outside*			
At least once a week	324 (63.2)	177 (62.5)	0.724
Less than once a week and at least once a month	107 (20.9)	55 (19.4)	
Less than once a month	82 (16.0)	51 (18.0)	
*Taking medicine*			
Independent	379 (73.9)	195 (68.9)	0.134
Needs help	134 (26.1)	88 (31.1)	
*Money management*			
Independent	377 (73.5)	202 (71.4)	0.545
Partially needs help	100 (19.5)	55 (19.4)	
Totally needs help	36 (7.0)	26 (9.2)	
*Shopping*			
Independent	170 (33.1)	88 (31.1)	0.556
Needs help	343(66.9)	195 (68.9)	
*Cooking*			
Independent	258 (50.3)	126 (44.5)	0.296
Partially needs help	86 (16.8)	53 (18.7)	
Totally needs help	169 (32.9)	104 (36.7)	
**Preventive care service utilization**			
Used	433 (84.4)	93 (32.9)	< 0.001
- Preventing long-term care need and comprehensive livelihood support services	333 (64.9)	73 (25.8)	< 0.001
- Day services or day care services	85 (16.6)	12 (4.2)	< 0.001
- Home-visit services ^b)^	24 (4.7)	6 (2.1)	0.070
- Others	19 (3.7)	3 (1.1)	0.029

^(a)^*p*-value: χ^2^-test (*p* < 0.05). ^(b)^ Home-visit services included home help services, home-visit bathing services, home-visit nursing, home-visit rehabilitation, and physician/dentist home visit

Abbreviations: IADL: Instrumental Activities of Daily Living

The percentage and incidence of care needs level deterioration among older adults who implemented housing adaptations are shown in Table 3. Older adults who implemented housing adaptations were less likely to have care needs deterioration, compared to those who did not (30.4% vs. 43.9%, p = 0.001), during the average follow-up of 15.7 months for implemented housing adaptations and 13.7 months for not implemented housing adaptations. Similarly, the incidence of care needs level deterioration was lower in older adults who implemented housing adaptations (19.3/1000 person-month), compared to those who did not (31.9/1000 person-month). The numbers of older adults who died or moved out of the municipality were fewer than 10 for each group, except for 16 older adults who died during the study period in those who did not implement housing adaptation.

**Table 3:** Care needs level deterioration among older adults with care support levels according to housing adaptations

	Care needs level deterioration n (%)	Follow-up period (month) (Mean ± SD)	Incidence of care needs level deterioration (/1000 person-month)	Model 1		Model 2	
				aHR	95%CI	aHR	95%CI
Housing adaptations							
Not implemented	225 (43.9)	13.7 ± 8.4	31.9	ref		ref	
Implemented	86 (30.4)	15.7 ± 8.1	19.3	0.69	0.51–0.93		
- Not maximum cost group	64 (33.7)	15.4 ± 8.5	21.8			0.78	0.57–1.07
- Maximum cost group	22 (23.7)	16.3 ± 7.3	14.4			0.51	0.31–0.82

aHR: adjusted hazard ratio; 95% CI: 95% confidence interval

The exposures in models 1 and 2 were implementation of any housing adaptations and implementation of housing adaptations divided whether the cost was maximum (USD1,345–1,513) or not maximum cost (< USD1,345), respectively. Models were adjusted for age, sex, household income, certification levels, cognitive function, instrumental activities of daily living, and use of preventive care services

**Fig. 1:**
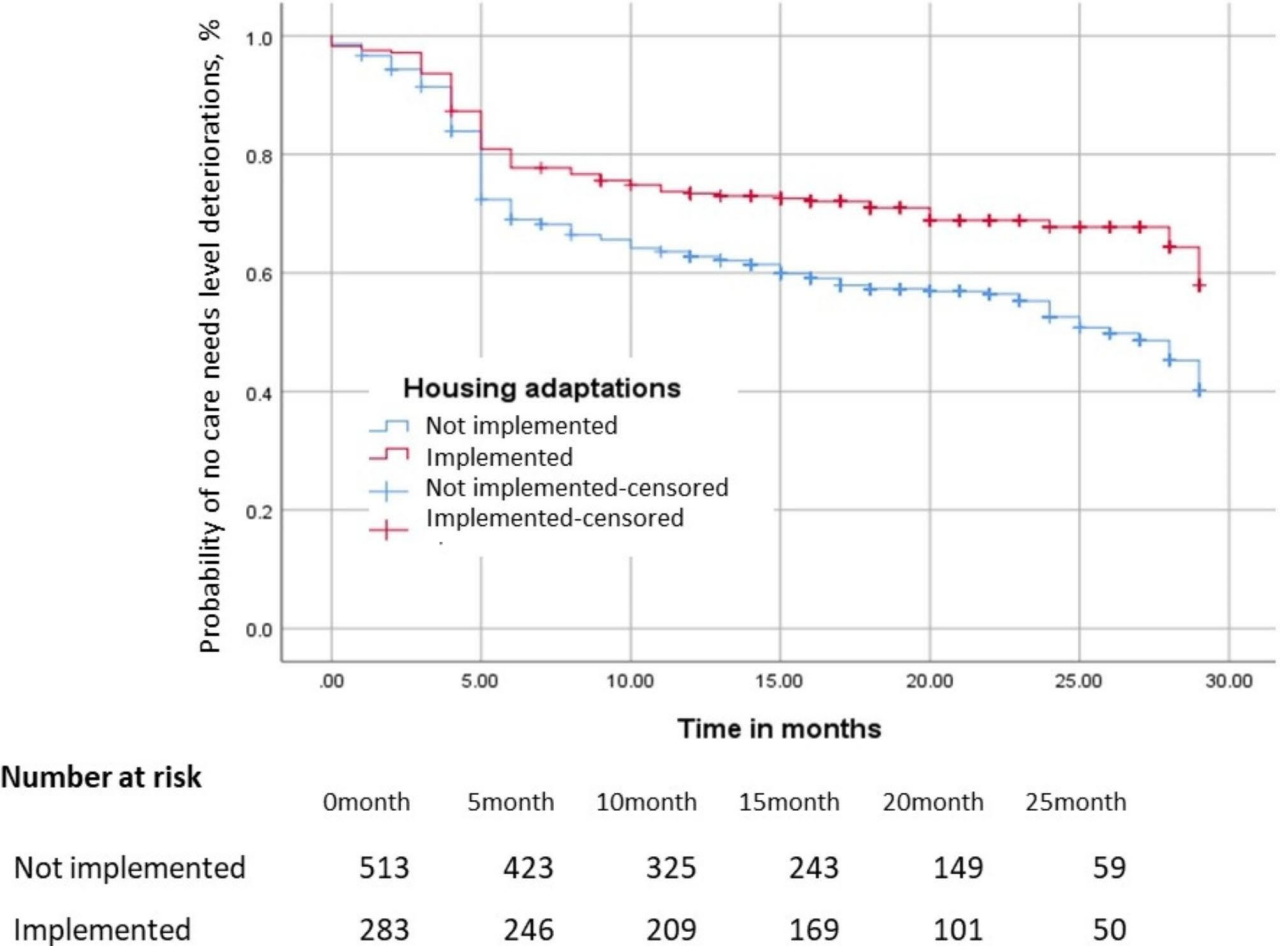
Kaplan − Meier plots of older adults’ care needs level deterioration according to housing adaptations

Kaplan–Meier plots of care need level deterioration according to the implementation of housing adaptations are shown in Fig. 1. The aHR of care needs level deterioration among older adults who implemented housing adaptations was 0.69 (95% CI: 0.51–0.93). In the analysis according to the total costs of housing adaptations being maximum or not, the aHRs for not maximum cost and maximum cost groups were 0.78 (95% CI: 0.57–1.07) and 0.51 (95% CI: 0.31–0.82), respectively, compared with those without housing adaptation. The double logarithms plot showed the survival function proportionally. In a sensitivity analysis of the implementation of housing adaptations in the first 12 months of care support certification compared with that at six months, the estimates were similar to those of the main results.

## Discussion

To the best of our knowledge, this is the first study to examine the effects of housing adaptations on care needs level deterioration among older adults with frailty in Japan. Older adults who implemented housing adaptations showed a lower risk of care needs level deterioration; in particular, those whose housing adaptation costs were maximum showed less frequent deterioration in their care needs. Our findings suggest that implementing housing adaptations for older adults with frailty was associated with lower risk of the further deterioration of care needs.

Our finding would add new evidence to that generated from a previous study [9]. The previous study’s sample included more dependent individuals with all care needs levels, including those who need caregiving throughout the day [28]. However, the purpose of housing adaptations differs according to disability levels [12]. Although housing adaptations for older adults with care support levels mainly focus on promoting capacity-enhancing behaviors, the main purpose for older adults with care needs levels is removing barriers to providing caregiver support. The differences in purpose mean that the outcomes which we should assess are also different, and a sample with a wide range of care needs levels might have obscured the effect of housing adaptations. Older adults with frailty are vulnerable to adverse outcomes, [13] and therefore an important target for preventing care needs level deterioration. Housing adaptations could promote capacity-enhancing behaviors, maintain ADL, [5] and might have prevented the extension of total care time for older adults with frailty.

The maximum cost group was a significantly lower incidence of care needs level deterioration, but it did not reach the statistical significance for the not maximum cost group. The lower cost of housing adaptations might not have met these applicants’ needs. However, medical or long-term care professionals assess these applicants’ needs before housing adaptations are implemented under Japan’s long-term care insurance system, [3] and this pre-assessment system will lower the risk of missing the needs. Rather than that, this result might be because the not maximum cost groups might be too weak as an intervention for building environments. Although differences existed in who is eligible for housing adaptations, the Japanese government set a very low cost for housing adaptations compared to other countries (e.g., the maximum cost is GBP 30,000 (USD 40,766) in the UK [29] and EUR 4,000 (USD 4,360) in Germany [30]). About 30% of older adults using housing adaptations in Japan exceed the upper limit by themselves with 100% out-of-pocket payments [31]. Housing adaptations with at least the maximum cost of this service may prevent increasing care needs. The dose-response relationships between higher housing adaptation costs and lower relative risks of care needs level deterioration might exist, but further studies are needed to assess the fulfillment of clients’ needs and the causal relationships.

This study has three major limitations. First, it is impossible to detail the contents of housing adaptation, such as the types of housing adaptations based on our claims data. We speculated that most housing adaptations were installing handrails according to a previous study [31]. Our research suggested that housing adaptation amounts were also related to the care needs levels; therefore, in further research, we should examine the representative contents of housing adaptations and their amount to determine the effects of housing adaptations. Second, we could not exclude the possibility of the existence of residual confounding factors affecting the study results. We could not adjust other confounding factors such as malnutrition or social frailty [32] because older adults who did not implement housing adaptation services were more likely to use preventive care services. Additionally, we could not adjust for medical backgrounds (e.g., heart failure, musculoskeletal disorders) as potential confounding factors because the medical data were unavailable. Although the care needs certification data includes specific medical procedures such as the use of oxygen therapy, we did not include these because the number of study subjects was limited. Moreover, we could not identify older adults who were admitted to a hospital and they were regarded as not having experienced care needs level deterioration during the follow-up period. Future research requires collaboration between medical and long-term care claims data. Third, although the baseline period was six months, some individuals might implement housing adaptations even after this period. Therefore, the results of this study mainly pertain to those who implemented housing adaptations either immediately or soon after care support certification. Moreover, we performed follow-up assessments only for two years, which may be too short a period to evaluate the effect of long-term care services; therefore, further research is needed to examine the effect of housing adaptations for older adults with disabilities using longer evaluation periods. Despite these limitations, our results revealed the association of housing adaptations with the required time for care by using representative data from one municipality in Japan.

## Conclusion

This study offers novel insights into the housing adaptations on the prevention of care needs level deterioration among older adults with frailty. Policymakers and health professionals need to deliver housing adaptations for this population when appropriate. Additionally, further research is needed to examine the longer-term effects of housing adaptations, considering the fulfillments of clients’ needs and the causal relationships.

## Data Availability

All relevant data underlying this study are owned by the municipality’s project. There is an agreement between the municipality and the Dia Foundation for research on ageing societies. The agreement stipulates the following; this municipality does not allow the authors to use the data for any purpose other than this project or provide them to anyone other than the study members without permission from this municipality. Researchers interested in the data used here should contact Dia Foundation for Research on Ageing Society.

## Abbreviations

ADL: Activities of daily living
BADL: Basic activities of daily living
IADL: Instrumental activities of daily living
aHR: Adjusted hazard ratio
95% CI: 95% confidence intervals

## References

[1] Thorstensen-Woll C, Buck D, Naylor C. Homes, health and COVID-19: Centre for Ageing Better. Published 2020. Accessed April 20, 2023. https://ageing-better.org.uk/sites/default/files/2021-08/Homes-health-and-COV19-poor-quality-homes.pdf.

[2] Chandola T, Rouxel P (2022). Home modifications and disability outcomes: a longitudinal study of older adults living in England. Lancet Reg Health - Europe.

[3] Tsuchiya-Ito R, Iwarsson S, Slaug B. Environmental Challenges in the Home for Ageing Societies: a comparison of Sweden and Japan. J Cross Cult Gerontol. 2019;34(3). 10.1007/s10823-019-09384-6. 10.1007/s10823-019-09384-6 31506755

[4] Hwang E, Cummings L, Sixsmith A, Sixsmith J. Impacts of Home Modifications on Aging-in-Place. 2011;25(3):246–57. 10.1080/02763893.2011.595611

[5] Carnemolla P, Bridge C (2018). A scoping review of home modification interventions – mapping the evidence base. https://doiorg.

[6] Keall MD, Pierse N, Howden-Chapman P (2015). Home modifications to reduce injuries from falls in the home injury prevention intervention (HIPI) study: a cluster-randomised controlled trial. Lancet.

[7] Boström L, Chiatti C, Thordardottir B, Ekstam L, Fänge AM. Health-related quality of life among people applying for housing adaptations: Associated factors. Int J Environ Res Public Health. 2018;15(10). 10.3390/ijerph15102130. 10.3390/ijerph15102130 PMC6211042 30262784

[8] Carnemolla P, Bridge C. Housing design and community care: how home modifications reduce care needs of older people and people with disability. Int J Environ Res Public Health. 2019;16(11). 10.3390/ijerph16111951. 10.3390/ijerph16111951 PMC6604004 31159396

[9] Mitoku K, Shimanouchi S (2014). Home modification and prevention of frailty progression in older adults: a japanese prospective cohort study. J Gerontol Nurs.

[10] Powell J, Mackintosh S, Bird E, Lge J, Garrett H, Roys M. The role of home adaptations in improving later life. Centre for Ageing Better. Published 2017. Accessed March 8, 2022. https://ageing-better.org.uk/publications/role-home-adaptations-improving-later-life.

[11] Crowell NA, Sokas RK (2020). Safe at home: a quasi-experimental evaluation of a municipal intervention to prevent falls among low-income elderly. J Health Care Poor Underserved.

[12] WHO. World report on ageing and health. Published 2015. Accessed April 6., 2022. https://apps.who.int/iris/handle/10665/186463.

[13] Searle SD, Mitnitski A, Gahbauer EA, Gill TM, Rockwood K (2008). A standard procedure for creating a frailty index. BMC Geriatr.

[14] Ministry of Health, Labour and Welfare. Legislation related to long-term care certifications [Youkaigonintei ni kakaru hourei]. Accessed July 24., 2022. https://www.mhlw.go.jp/stf/seisakunitsuite/bunya/hukushi_kaigo/kaigo_koureisha/nintei/gaiyo4.html.

[15] Cabinet office, Government of Japan. White Paper 2016-The Current Situation of Aging-[Heisei28nen Kourei Shakai Hakusyo]. Published 2016. Accessed Aug 22., 2023. https://www8.cao.go.jp/kourei/whitepaper/w-2016/html/gaiyou/s1_1.html.

[16] Kawagoe S, Tsuda T, Doi H (2013). Study on the factors determining home death of patients during home care: a historical cohort study at a home care support clinic. Geriatr Gerontol Int.

[17] Morgan CJ (2019). Landmark analysis: a primer. J Nucl Cardiol.

[18] Suissa S (2004). Inhaled steroids and mortality in COPD: Bias from unaccounted immortal time. Eur Respir J.

[19] Tsuchiya-Ito R, Hamada S, Slaug B, Ninomiya A, Uda K, Ishibashi T. Implementation and costs of housing adaptations among older adults with different functional limitations in Japan. BMC Geriatrics 2022;22(1):1–13. 10.1186/S12877-022-03100-9. 10.1186/s12877-022-03100-9 PMC9123739 35596138

[20] Bank of Japan. Tables of key time series statistics [in Japanese]. Published 2022. Accessed January 30., 2022. https://www.stat-search.boj.or.jp/ssi/mtshtml/fm08_m_1.html.

[21] Rautio N, Heikkinen E, Heikkinen RL (2001). The association of socio-economic factors with physical and mental capacity in elderly men and women. Arch Gerontol Geriatr.

[22] Béland F, Zunzunegui MV (1999). Predictors of functional status in older people living at home. Age Ageing.

[23] Ho SC, Woo J, Yuen YK, Sham A, Chan SG. Predictors of mobility decline: the Hong Kong old-old study. J Gerontol A Biol Sci Med Sci. 1997;52(6). 10.1093/GERONA/52A.6.M356. 10.1093/gerona/52a.6.m356 9402942

[24] Ohn J, Ynch WL, Aplan EAK, Arah S, Hema JS. Cumulative impact of sustained economic hardship on physical, cognitive, psychological, and social functioning. NEJM 2009;337(26):1889–95. 10.1056/NEJM199712253372606 10.1056/NEJM199712253372606 9407157

[25] Lawton MP, Brody EM (1969). Assessment of older people: self-maintaining and instrumental activities of daily living. Gerontologist.

[26] Nakai Y (2014). A study of persons who do not use care services of public longterm care insurance system [in Japan]. Doshisha policy and management review.

[27] Ikeda S. The current situation of care needs certification and utilization of long-term care services [in Japanese]. Published 2010. Accessed April 6, 2022. https://www.mhlw.go.jp/shingi/2010/01/dl/s0115-9j.pdf.

[28] Ministry of Health, Labour and Welfare. Long-term care need certification in long-term care insurance system [in Japanese]. Accessed April 5., 2022. https://www.mhlw.go.jp/topics/kaigo/kentou/15kourei/sankou3.html.

[29] Mackintosh S, Smith P, Garrett H, Davidson M, Morgan G, Russell R. Disabled Facilities Grant (DFG) and Other Adaptations -External Review; 2018. Accessed 22 Aug 2023. https://www.gov.uk/government/publications/disabled-facilities-grant-and-other-adaptations-external-review.

[30] Bundesministerium für Gesundheit. Measures to improve the living environment [Wohnumfeldverbessernde Maßnahmen]. Accessed July 24, 2022. https://www.bundesgesundheitsministerium.de/leistungen-der-pflege/wohnumfeldverbessernde-massnahmen.html.

[31] Ishikawa Y, Koike K (2008). Results of the fact-finding study of home renovation utilizing nursing care insurance [in Japanese]. Josai Int Univ Bull.

[32] Yamada M, Arai H (2018). Social Frailty Predicts Incident Disability and Mortality among Community-Dwelling Japanese older adults. J Am Med Dir Assoc.

[33] Tsutsui T, Muramatsu N (2005). Care-needs certification in the long-term care insurance system of Japan. J Am Geriatr Soc.

[34] Ito T, Mori T, Takahashi H (2021). Prevention services via public long-term care insurance can be effective among a specific group of older adults in Japan. BMC Health Serv Res.

